# Whole‐genome re‐sequencing provides key genomic insights in farmed Arctic charr (*Salvelinus alpinus*) populations of anadromous and landlocked origin from Scandinavia

**DOI:** 10.1111/eva.13537

**Published:** 2023-02-27

**Authors:** Fotis Pappas, Khrystyna Kurta, Tytti Vanhala, Henrik Jeuthe, Ørjan Hagen, José Beirão, Christos Palaiokostas

**Affiliations:** ^1^ Department of Animal Breeding and Genetics Swedish University of Agricultural Sciences Uppsala Sweden; ^2^ Aquaculture Center North Kälarne Sweden; ^3^ Faculty of Bioscience and Aquaculture Nord University Bodø Norway

**Keywords:** Arctic charr, genetic diversity, selective sweeps, whole‐genome resequencing

## Abstract

Arctic charr (*Salvelinus alpinus*) is a niche‐market high‐value species for Nordic aquaculture. Similar to other salmonids, both anadromous and landlocked populations are encountered. Whole‐genome re‐sequencing (22X coverage) was performed on two farmed populations of anadromous (Sigerfjord; *n* = 24) and landlocked (Arctic Superior; *n* = 24) origin from Norway and Sweden respectively. More than 5 million SNPs were used to study their genetic diversity and to scan for selection signatures. The two populations were clearly distinguished through principal component analysis, with the mean fixation index being ~0.12. Furthermore, the levels of genomic inbreeding estimated from runs of homozygosity were 6.23% and 8.66% for the Norwegian and the Swedish population respectively. Biological processes that could be linked to selection pressure associated primarily with the anadromous background and/or secondarily with domestication were suggested. Overall, our study provided insights regarding the genetic composition of two main strains of farmed Arctic charr from Scandinavia. At the same time, ample genomic resources were produced in the magnitude of millions of SNPs that could assist the transition of Nordic Arctic charr farming in the genomics era.

## 1 INTRODUCTION

Domestication of fish occurred more recently than terrestrial animals (Teletchea & Fontaine, 2014). Due to the booming of the aquaculture industry over the last four decades, substantial selective pressure has been applied to farmed fish, aiming to improve primarily traits of economic value. As is the case with natural selection, artificial selection tends to leave footprints in the genome known as selection signatures that usually harbour genes influencing key fitness traits (López et al., 2015). Therefore, the detection of selection signatures can assist in identifying causative genes for such traits and elucidate the underlying molecular mechanisms (Ghoreishifar et al., 2020). Notably, a substantial knowledge gap exists about the genetic architecture of traits targeted by artificial selection (Regan et al., 2021), often attributed to the polygenic nature of many of those traits (Robledo et al., 2018).

Moreover, as farmed fish are characterized by high levels of fecundity, short‐term production goals can be reached using a relatively low number of broodfish, which runs the risk of rapid inbreeding accumulation (Wang et al., 2002). In the typical scenario where intensive selection is applied and guided solely by the breeding candidate's phenotype, the problem can further exacerbate and potentially lead to inbreeding depression (You & Hedgecock, 2019). Nevertheless, even in the scenario where pedigree recordings are used to avoid crossings between closely related animals, only an average estimate of inbreeding is possible, which also fails to capture the within‐family relatedness component (Meuwissen et al., 2020). Furthermore, because the animals that constitute the base population are assumed to be unrelated, the overall inbreeding level is usually underestimated (Howard et al., 2017).

Advancements in genomics allow the application of whole‐genome sequencing technologies for in‐depth studies on genetic diversity, detection of selection signatures and estimation of genome‐wide inbreeding (Alemu et al., 2021; Cádiz et al., 2020). Simultaneously, a plethora of analytical methods has been fine‐tuned for harnessing the enormous amounts of genomic data that modern sequencers output. Regarding the detection of selection signatures, most analytic methods rely either on population differentiation metrics (e.g. *F*_ST_ index), on the expected allele frequency distribution under different selection scenarios (e.g. Tajimas's *D* index) or on haplotype statistics that incorporate linkage disequilibrium (LD) patterns like XP‐EHH (Sætre & Ravinet, 2019). Nevertheless, it is often the case that those statistics do not return consistent results, which is partly explained by the fact that no analytic method can capture the entire spectrum of selection signature types (Hohenlohe et al., 2010; Lotterhos & Whitlock, 2015). Ensemble approaches like the de‐correlated composite of multiple metrics (DCMS), where different selection signature statistics are aggregated under a unified metric, can result in higher prediction accuracy and reliability (Ma et al., 2015).

Arctic charr (*Salvelinus alpinus*) is a high‐value species in the Arctic region (Helgadóttir et al., 2021). Found in both anadromous and freshwater forms (Berg & Berg, 1993), has inhabited a diverse range of Arctic ecosystems with some glacial relic stocks in the European Alps (Klemetsen et al., 2003; Kottelat & Freyhof, 2007; Tiberti & Splendiani, 2019). Furthermore, due to its considerable capacity for growth in cold waters, Arctic charr is an ideal species for farming in the Arctic region. Not surprisingly, Arctic charr is farmed in Nordic countries (Iceland, Sweden, Norway) and Canada (Sæther et al., 2013).

Even though in terms of production volume, Arctic charr ranks considerably lower than the industry's leading salmonid species like Atlantic salmon (*Salmo salar*) and rainbow trout (*Oncorhynchus mykiss*), ample margin exists for expansion, constituting the species a strong candidate for diversification (Palaiokostas et al., 2020). However, unlike the salmonids mentioned above, where genomic information is routinely applied (Houston et al., 2020), genomic‐based studies in Arctic charr are still relatively limited.

Recent studies with a primarily ecological‐evolutionary focus have generated genomic resources for Arctic charr in the form of a medium‐density SNP array (Nugent et al., 2019) and studied genetic diversity and adaptation to environmental factors in Canadian populations of wild Arctic charr using genotyping by sequencing and whole‐genome re‐sequencing (Dallaire et al., 2021; Kess et al., 2021). On the other hand, limited research based on genomic technologies has been conducted in farmed Arctic charr (Gudbrandsson et al., 2016). During the last couple of years, the usage of genomic information for selective breeding was assessed in Swedish Arctic charr (Palaiokostas et al., 2022; Pappas & Palaiokostas, 2021). Nevertheless, the aforementioned studies used genomic tools of relatively low genotyping density.

In the current study, we performed whole‐genome re‐sequencing in farmed Arctic charr from Norway and Sweden of anadromous and landlocked origin respectively. Knowledge about the physiological and social‐behavioural mechanisms of migration in salmonid species is increasing (Berdahl et al., 2016; Keefer & Caudill, 2014). Nevertheless, examining the genetic differences between charr populations of landlocked and anadromous backgrounds provides an opportunity to study those mechanisms at the genome level. As mentioned above, natural selection tends to leave genetic footprints. Given information about the biological functions linked with such genomic regions and the possible ecological context of selective pressure affiliated to anadromy, valuable knowledge can be obtained. Simultaneously, the genetic diversity status of the two populations was studied in depth resulting in information that could be used for optimizing their management.

## 2 MATERIALS AND METHODS

### 2.1 Background information of the studied populations

Arctic charr from the Swedish national breeding programme and a commercial farm in Norway was used in our study. Briefly, the Swedish breeding programme has been operating since the early 80s by Aquaculture Centre North (ACN) in facilities located in central Sweden. The base population was formed from animals originating from the Swedish lake Hornavan and no external breeders have been introduced ever since (Eriksson et al., 2010; Nilsson et al., 2010). Currently, the breeding programme is in the ninth generation. The breeding design involves separately fertilizing the eggs of two females from one male. Thereafter individual families are reared in separate incubation trays until hatching, upon which they are transferred in separate 1 m^3^ tanks until they reach a size suitable for marking with passive integrated transponders (PIT tags). Following tagging, the animals are communally reared. Overall, the number of full‐sib families per generation ranges between 45 and 125 (Nilsson et al., 2010; Palaiokostas et al., 2021). Throughout the production cycle, the animals are reared in freshwater and selection decisions focus primarily on improving growth. Available information suggests that substantial growth gains have been obtained so far (Carlberg et al., 2018). Simultaneously, breeding amongst closely related animals is avoided (Palaiokostas et al., 2022). For the needs of the current study, 24 broodfish (12 males; 12 females) from year‐classes 2013 and 2017 were used (equal number of males and females from each year‐class). Out of those animals, 20 originated from different full‐sib families, while no pedigree records were available for four animals from the 2013 year‐class.

The second population came from a commercial farm located in Sigerfjord, Norway. The founders of the Norwegian population originated mainly from the Hammerfest strain (~60%–70%), while the remaining part came from Svalbard (Norway) and Iceland (personal communication with Sigerfjord Fisk AC, January 2021). Notably, the Hammerfest strain can be reared in full‐strength sea water and is considered anadromous, while the bulk production of Icelandic Arctic charr takes place in brackish water (Arnason et al., 2014; Arnesen et al., 1993). Own egg production started in 1995, and the breeding population consists of 700–800 females and 150 males, with seven generations recorded so far in captivity. No pedigree records are kept in this setting, and breeding candidates of every generation undergo selection based on their own phenotype (mass selection), which is primarily focused on growth. Finally, rearing takes place in brackish water of differing salinity strength depending on the season (e.g. lower salinity strength during summer months). In the current study, 24 broodfish (12 males; 12 females) from the Sigerfjord population were used. Since no pedigree recordings were available, the age and the relationship level of those animals were unknown.

### 2.2 Genomic DNA extraction—sequencing

Genomic DNA was extracted from fin‐clip samples of 48 animals following the standard protocol for solid tissue using the Quick‐DNA Miniprep Plus Kit (Zymo Research, USA). In summary, the DNA was digested using a lysis solution containing 10 μL of proteinase K (20 mg/mL), 95 μL of Solid Tissue Buffer, and 95 μL of water, at 55°C for 3 h. Thereafter, lysates were centrifuged at 13,000 ***g*** for 1 min to remove insoluble debris. The supernatant (~200 μL) was combined with the manufacturer's binding buffer (2 volumes of supernatant), loaded onto spin columns, and then centrifuged at 13,000 ***g*** to bind the DNA. Subsequent wash steps were performed according to the manufacturer's protocol. Elution was done with a pre‐heated (70°C) elution buffer (10 mM Tris‐Cl, pH 8.5) and 5 min incubation at room temperature before centrifugation. The elution was repeated twice for a total volume of ~50 μL.

The purity of the extracted DNA was assessed by spectrometry using a NanoDrop 8000 (Thermo Fisher). The 260 nm/280 nm and 260 nm/230 nm ratios were measured to evaluate DNA purity. DNA concentration was measured on Qubit 2.0 fluorometer using the dsDNA Broad Range Assay Kit (Invitrogen, Life Technologies). Furthermore, the integrity of the extracted DNA was assessed by agarose gel (1.5%) electrophoresis using the SYBR Safe DNA Stain (Invitrogen, Life Technologies) and was visualized using the ChemiDoc™ Touch Imaging System (Bio‐Rad).

Thereafter, DNA samples were sent to the National Genomics Infrastructure Center in Uppsala, Sweden. The TruSeq DNA PCR‐free kit (Illumina) was used for library preparation, followed by whole‐genome sequencing in an Illumina NovaSeq6000 instrument using three lanes of two S4 v1.5 flow cells with a read setup of 2 × 150 cycles.

### 2.3 Quality control, trimming and read mapping

The sequence data quality of each individual sample was assessed with FASTQC v0.11.8, while MultiQC (Ewels et al., 2016) v1.8 was used to produce a single quality report for all the samples. FASTP v0.22.0 (Chen et al., 2018) was used to trim adapter‐oligomeric sequences and filter out reads with a Phred quality score of <25. The remaining reads were aligned to *Salvelinus* sp. genome assembly (ASM291031v2) using Bowtie2 v2.4.4 (Langmead & Salzberg, 2012), while Samtools v1.13 (Danecek et al., 2021) was used to sort the alignments and convert the output to binary format (BAM files). It is worth noting that the reference genome used in our study was not from *Salvelinus alpinus*, but from the closely related species *Salvelinus malma* or a hybrid of the two.

### 2.4 SNP calling, filtering and genotype metrics

Following the addition of read group tags (https://github.com/ekg/bamaddrg), SNP detection was performed using FreeBayes v1.3.5 (Garrison & Marth, 2012) with 40 threads (Tange, 2018) to obtain biallelic SNP genotypes in variant call format (VCF). Additional data filtering of the marker set was carried out with VCFtools v0.1.16 (Danecek et al., 2011). In particular, SNPs of the nuclear genome with minor allele frequency above 5% and call‐rate of 100% were retained for downstream analysis. SNP density in 100 kb non‐overlapping windows and mean read depth per site were estimated using the ‐‐*SNPdensity* and ‐‐*site‐mean‐depth* functions of VCFtools and visualized with the R package circlize v0.4.10 (Gu et al., 2014). Finally, heterozygosity metrics were calculated with the ‐‐*het* function of the same suite.

### 2.5 Population structure, admixture and genomic relationships

Population structure was deciphered by principal component analysis (PCA) with PLINK v1.90b6.21 (Chang et al., 2015) after converting the VCF file to a PLINK‐readable file using ‐‐*plink*/VCFtools and performing linkage filtering (*r* ^2^ < 0.15, window = 50 kb, step = 10 kb). A subset of the previous dataset containing 108,903 SNPs (*r* ^2^ ≤ 0.01, window = 100, step = 10) was used in a model‐based analysis to assess ancestry. This part was carried out using ADMIXTURE v1.3.0 (Alexander et al., 2009). To determine the ‘optimal’ number of clusters (*K*) cross‐validation error scores were calculated for *K* values from one to five. Furthermore, in order to decipher the realized relationships amongst the studied populations a genomic relationship matrix was computed using the PLINK function *‐‐make‐rel* that implements the computation formula described by Yang et al. (2011). Finally, the Welch's *t*‐test (Welch, 1947) was employed to compare within‐stock genetic relatedness.

### 2.6 LD decay analysis and demographical history

The software popLDdecay v3.40 (Zhang et al., 2019) was used to calculate linkage disequilibrium (LD) in physical distances ranging up to 500 kb separately for the two populations. The same procedure was repeated separately for males and females to gain insights regarding potential recombination differences between the two sexes. Additionally, effective population sizes (*N*_e_) were computed for each population with SNeP v1.1 (Barbato et al., 2015) and demographic histories were inferred for the past 1000 generations. The latter was done using information from 10,000 randomly sampled SNP markers per pseudochromosome (unplaced contigs were not included in the analysis).

### 2.7 Runs of homozygosity and genomic inbreeding estimation

A lower MAF filter of 1.5% (on a metapopulation level) and exclusion of unplaced contigs were applied for a runs of homozygosity (ROH) analysis. The R (R core team, 2021) package detectRUNS v0.9.6 (Biscarini et al., 2018) was employed to identify ROH with a sliding window approach. Particularly, the window length was set to 2500 SNPs, the maximum gap at 10 kb and the minimum ROH length was defined at 1 Mb. Moreover, the maximum number of allowed ‘opposite’ genotypes was 150 per window (accounting for 6% of the window) and the number of minimum SNPs was set equal to window size as previously suggested (Meyermans et al., 2020; Selli et al., 2021) to decrease the occurrence rate of false negatives in the case of lower and higher thresholds. Overall, the detected runs were assigned to four different length classes: 1–2 Mb, 2–4 Mb, 4–8 Mb and 8–16 Mb. Furthermore, the yielded information allowed the calculation of inbreeding coefficients (*F*_ROH_) for each individual using the *Froh_inbreeding* function of detectRUNS. More specifically, the following formula was used:

(1) $$F_{\mathrm{ROH}}=\frac{1}{L_{\text{genome}}}\sum L_{\mathrm{ROH}}$$

where $L_{\mathrm{ROH}}$ is the physical length of a ROH and $L_{\text{genome}}$ is the total length of the reference genome.

### 2.8 Assembly and haplotype network analysis of mt‐genomes

Mitochondrial genomes were de novo assembled separately for each individual using GetOrganelle v1.7.5.0 (Jin et al., 2020). Five different *k‐mer* lengths (21 bp, 45 bp, 65 bp, 85 bp and 105 bp) were chosen, while the mitogenome sequence AF154851.1 (Doiron et al., 2002) of the *S. alpinus* host was used as seed. The yielded circular sequences were manually rotated so that all of them start with the same nucleotide. Following, the R package pegas v1.1 (Paradis, 2010) was used to perform multiple alignments with MUSCLE (Edgar, 2004; Paradis et al., 2004), detect mitochondrial haplotypes and construct a haplotype network. The consensus sequence of all 48 mitogenomes under study was extracted using UGENE v40.0 (Okonechnikov et al., 2012) after carrying out multiple alignments with the aforementioned algorithm.

### 2.9 Genome‐wide scans for identification of selection signatures

In order to detect with high probability genomic regions of having undergone recent positive selection, two methods were applied:

- Weir and Cockerham *F*‐statistic (*F*_ST_) (Weir & Cockerham, 1984) was calculated and averaged over 50 kb sliding windows with 25 kb overlap using the ‐‐*weir‐fst‐pop* function integrated in VCFtools. This allele frequency based, population differentiation metric is regarded to be a reliable indicator of selection signatures when scanning dense genotypic data (Ma et al., 2015; Willing et al., 2012).
- Following statistical haplotype phasing with Shapeit v2.r904 (Delaneau et al., 2012; Delaneau, Howie, et al., 2013; Delaneau & Marchini, 2014; Delaneau, Zagury, & Marchini, 2013; O'Connell et al., 2014), cross‐population extended haplotype homozygosity (XP‐EHH) (Sabeti et al., 2007) was calculated using the R package rehh v3.2.2 (Gautier et al., 2017) and the negative log_10_ of the obtained *p*‐values were averaged in the same sliding windows as in the *F*_ST_ analysis.

It has been previously suggested that combining different selection signature identification metrics can boost the statistical power of the analysis (Grossman et al., 2010, 2013; Ma et al., 2015). Therefore, we used the R package MINOTAUR (Verity et al., 2017) to calculate the de‐correlated composite of multiple metrics (Ma et al., 2015) for each sliding window containing at least 30 SNPs as follows:

(2) $${\text{DCMS}}_{w}=\sum_{t=1}^{n}\frac{\log\;\left(\frac{1-p_{\mathrm{wt}}}{p_{\mathrm{wt}}}\right)}{\sum_{i=1}^{n}\left|r_{\mathrm{it}}\right|}$$

where *p*_wt_ represents the *p*‐value of the *t‐*th statistic for window *w* and *r*_*it*_ corresponds to the correlation coefficient between the *i*th and the *t‐*th statistic (in our case *F*_ST_ and XP‐EHH; *n* = 2). For *F*_ST_, the *p*‐values were estimated by applying a right‐tailed test on the empirical distribution. Windows corresponding to the top 0.5% of the yielded DCMS empirical distribution were treated as candidate selective sweep regions.

### 2.10 Annotation and enrichment analysis of selection signals

A bed file was created for the windows selected from the analysis described above. The genomic regions were annotated following an analytical procedure similar to the one described in Baesjou and Wellenreuther (2021). In brief, using BEDTools v2.30.0 (Quinlan & Hall, 2010) we extended the genomic windows of interest by 10 kb on either side and intersected the resulting file with the GFF3 annotation file of the reference genome. Following, we retrieved the corresponding FASTA sequences and performed a DIAMOND v2.0.13 BLASTX (Altschul et al., 1990; Buchfink et al., 2015) search against the zebrafish (*Danio rerio*) protein database downloaded from Ensemble 105 (Howe et al., 2021) using an e‐value threshold of 10^−5^. Thereafter, the Ensemble Biomart tool (Smedley et al., 2015) was used to obtain the gene names and IDs associated with the yielded hits. The latter were then used as input in the database for annotation, visualization and integrated discovery (DAVID) v6.8 (Huang et al., 2009) to extract enrichment information from the Kyoto encyclopedia of genes and genomes (KEGG) (Kanehisa, 2019; Kanehisa et al., 2021; Kanehisa & Goto, 2000) and obtain UniProt (The UniProt Consortium, 2017) biological function terms (UP_KW_BIOLOGICAL_PROCESS).

For interpretation purposes, the Jaccard similarity coefficient was calculated for each pair of gene sets (subsets of the submitted gene list) enriched as different terms. This pairwise score expresses term‐similarity as the ratio between the gene counts in the intersection over the cardinality of the union of the two sets:

(3) $$J\left(A,B\right)=\frac{\left|A\cap B\right|}{\left|A\cup B\right|}$$

where ∩ and ∪ denote intersection and union respectively.

## 3 RESULTS

### 3.1 Whole‐genome sequencing and genotyping

**FIGURE 1:**
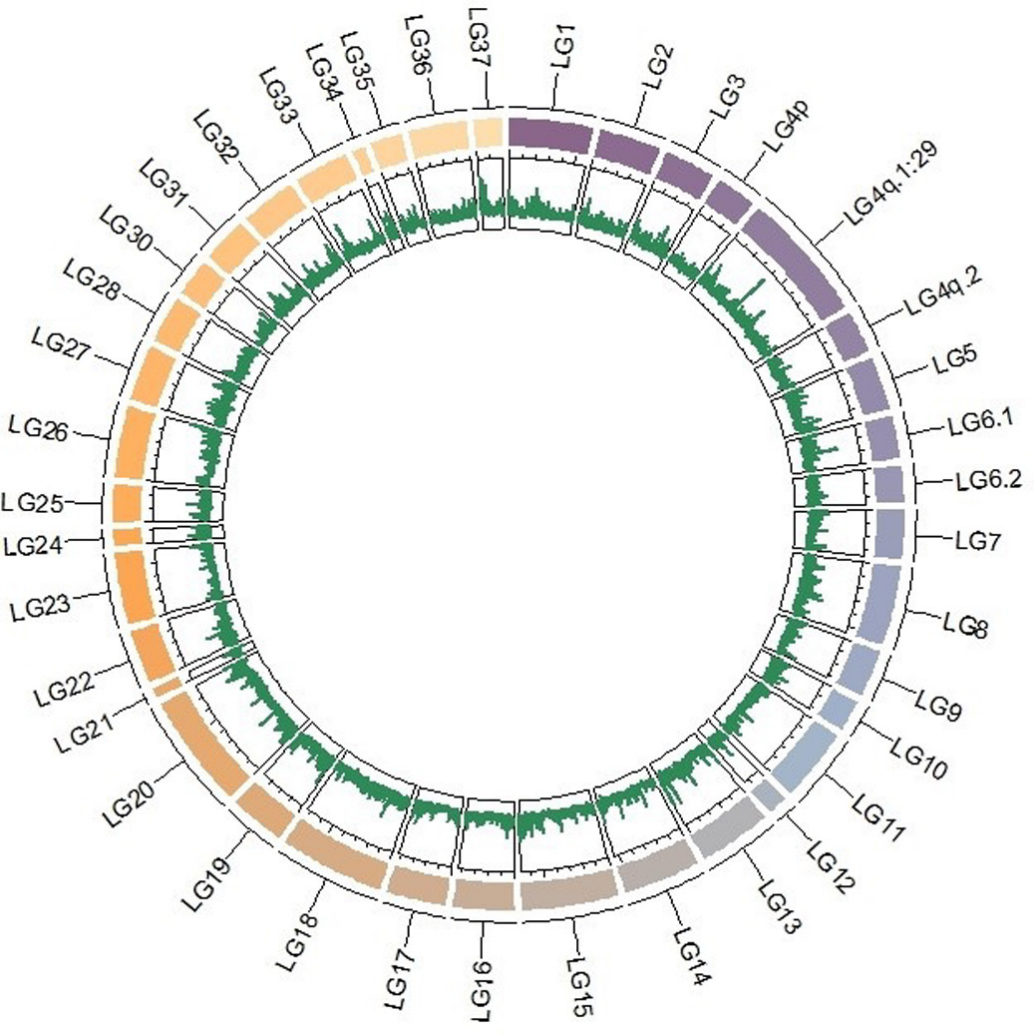
Circular visualization of the *Salvelinus* sp. reference assembly produced with R/circlize. Green line of the inner circle shows SNP density in the studied metapopulation along the physical positions of each pseudochromosome (linkage group). Density values range between 0.04 and 9.94 SNPs/b.

In total, whole‐genome re‐sequencing of 48 samples resulted in 15.57 billion reads with a mean GC content of 43.48%. Following QC, 14.47 billion reads passed the defined filters (~7% of reads were removed) and approximately 300 million reads on average were retained for each sample (File S2, S3). Read mapping against the *Salvelinus* sp. reference genome resulted in 84.79% mean overall alignment rate across samples. The downstream analysis of variant calling and marker set filtering (biallelic SNPs with MAF >0.05 and call‐rate = 1) resulted in a VCF file consisting of 6,505,695 SNPs. Overall, the mean genotypic density of this dataset was 2.59 SNPs/kb (Figure 1) with an average coverage of 22X. Finally, an observed heterozygosity of 29.42% and 29.47% was estimated for the Swedish and the Norwegian populations respectively.

### 3.2 Population structure, admixture and genomic relationships

**FIGURE 2:**
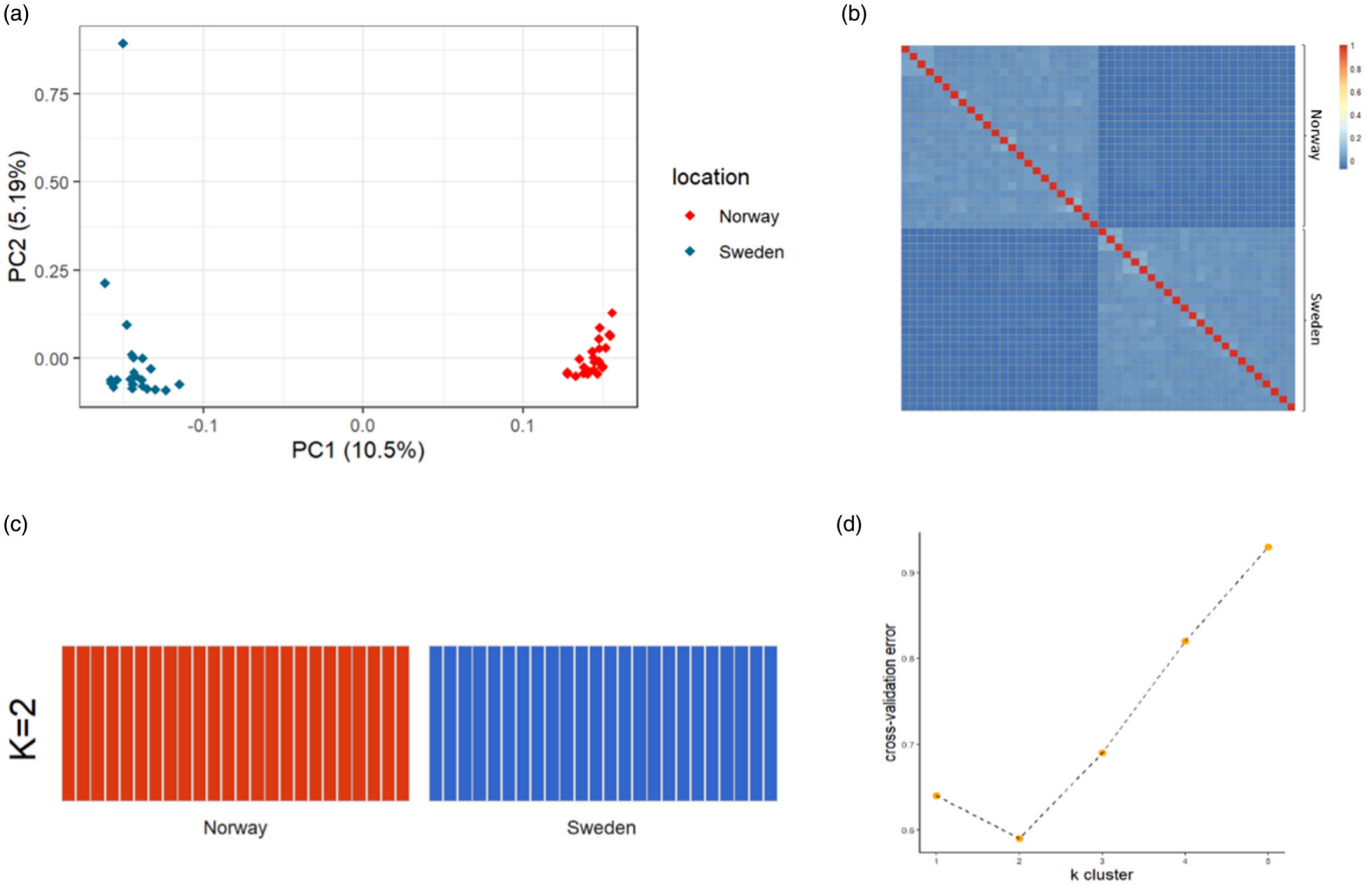
(a) Principal component analysis on LD‐pruned markers (*r* ^2^ *< 0.15, window = 50 kb, step = 10 kb*). The proportion of explained variance from each component is indicated inside parentheses next to their respective axis titles. (b) Heatmap visualizing the genetic relationship matrix (calculated with PLINK/*−−make‐rel*). (c) Admixture analysis on LD‐pruned markers (*r* ^2^ ≤ *0.01, window = 100, step = 10*) assigning individuals in clusters. Each single vertical bar represents an individual charr. The same colour in different individual charr suggests that they belong to the same cluster. (d) Cross‐validation errors for 1–5 clusters in the admixture analysis.

Principal component analysis using LD pruned markers (1,638,992 SNPs retained) provided insights regarding population structure by performing dimensionality reduction (Figure 2a) with the first (PC1) and second (PC2) principal components explaining 10.50% and 5.19% of the variance respectively. The two populations segregated along PC1, with the data points corresponding to the Swedish nucleus being more sparsely ordinated on both PC1 and PC2.

A clear discrimination between the two populations was observed also through the obtained genomic relationship matrix (Figure 2b). Furthermore, genetic relationships within population were higher than those across populations. The highest observed genetic relationship was 0.0895 and corresponded to a pair of charrs from the Swedish population, while no significant difference for within‐stock genomic relatedness was found between the Swedish and Norwegian charr. Finally, admixture results based on LD‐pruned SNPs (108,903 SNPs; *r* ^2^ ≤ 0.01, window = 100, step = 10) further supported the above findings since no hybridization of the two stocks was evident by suggesting that the ‘optimal’ number of clusters was two (Figure 2c,d).

### 3.3 LD decay and effective population size trends

**FIGURE 3:**
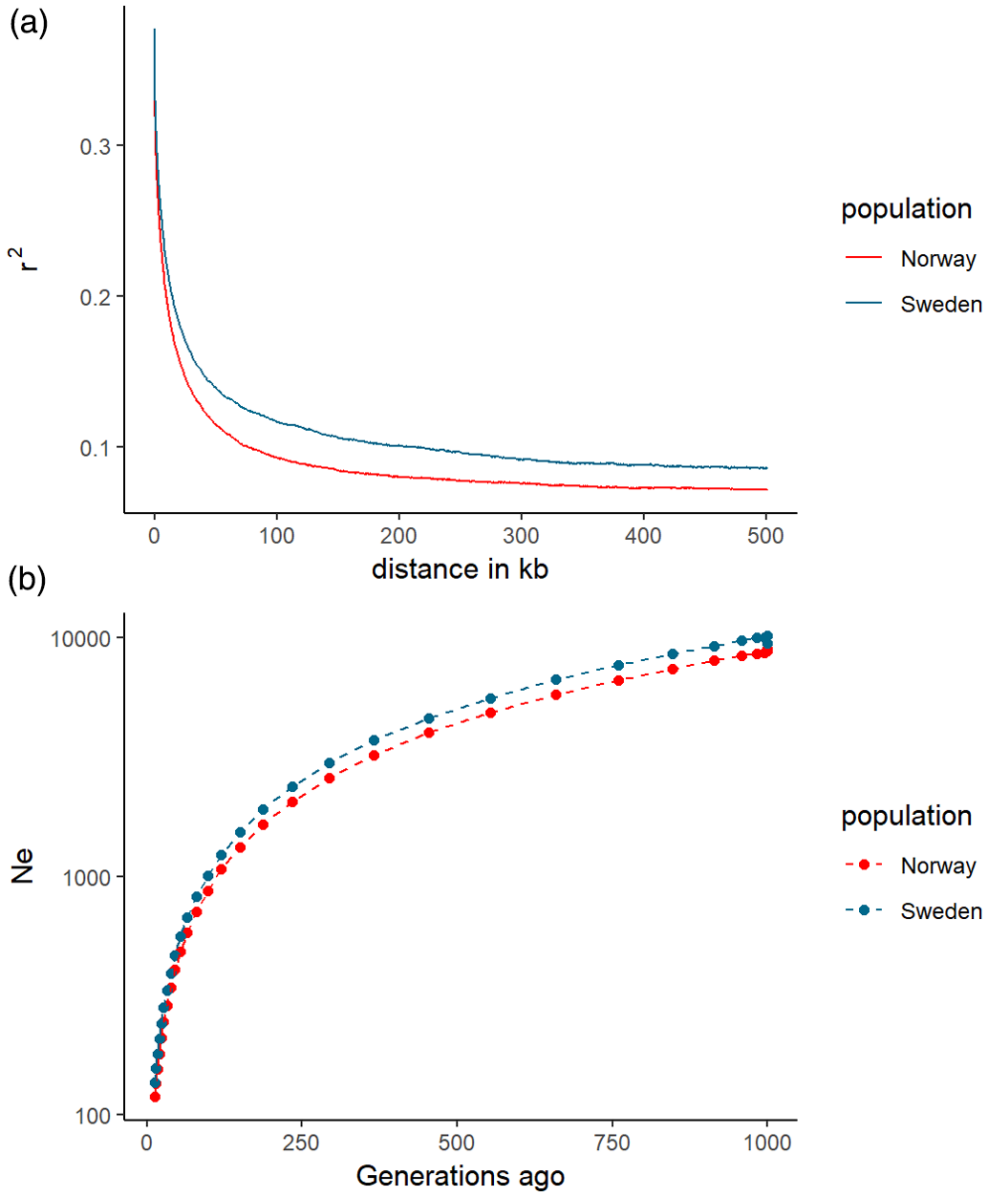
Trendlines: (a) *r* ^2^ of linkage disequilibrium by physical distance (in kb) for the two studied populations. (b) historical effective population size (*N*_e_) estimates for past generations spanning from 13 to 1000 generations ago. Vertical (*N*_e_) axis has undergone a log_10_ transformation. Estimates and trend lines are plotted in different colours for the two populations.

Mean *r* ^2^ values of LD decreased by physical distance between SNP pairs with the observed decay found to be more rapid in the Norwegian population (Figure 3a) that displayed an average *r* ^2^ of 0.09 at 100 kb compared to 0.12 for the Swedish one. Furthermore, no differences were found in LD decay between males and females of either population.

Historical demographic trends were visualized by plotting the estimated effective population size *N*_e_ (vertical axis is log_10_ transformed) over the past 1000 generations (Figure 3b). A similar, descending pattern through time was apparent for both populations with estimates for the Swedish population being slightly, but stably higher than the one from Norway. Nevertheless, it is worth noting that those estimates were characterized by high standard errors (Tables S1 and S2).

### 3.4 Runs of homozygosity and genomic inbreeding

The ROH detection analysis uncovered long homozygous genomic regions for each studied individual. The marker set used for that purpose consisted of 7,207,847 biallelic SNPs (MAF >0.015, 100% call‐rate, no markers on unplaced contigs; Table 1). ROH count and average length were higher in the Swedish population for every length class except the mean ROH length in the 0–2 Mb class that was ~20 kb longer in the Norwegian Arctic charr population. No runs exceeding the lengths defined by class 8–16 Mb were detected in neither population.

**TABLE 1:** ROH count and average length per length class comparisons between the Swedish and the Norwegian population.

Length class	Number of detected ROHs		Average length (Mb)	
	Norway	Sweden	Norway	Sweden
0–2 Mb	749	1077	1.40	1.38
2–4 Mb	299	376	2.66	2.70
4–8 Mb	74	102	5.01	5.06
8–16 Mb	5	13	10.16	10.40

**FIGURE 4:**
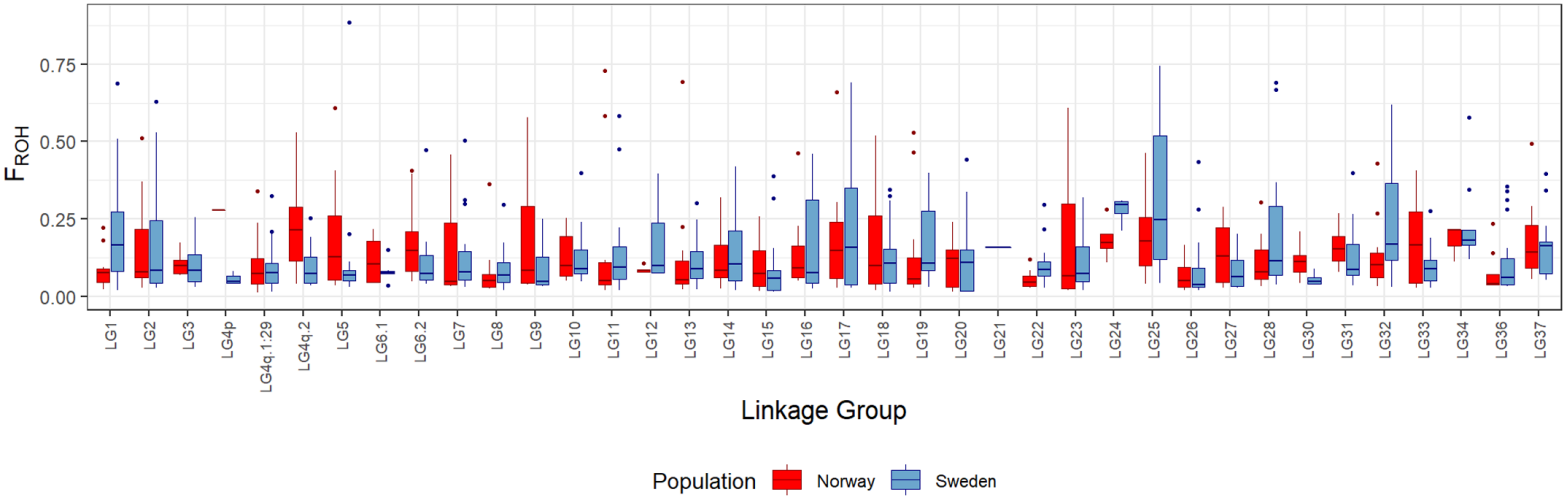
Boxplots illustrating the per‐pseudochromosome differences in *F*_ROH_ median values and variation between the studied Norwegian and the Swedish populations.

The estimated mean genomic inbreeding coefficient *F*_ROH_, was higher for the Swedish population (*F*_ROH_ = 8.66%) compared to the Norwegian (*F*_ROH_ = 6.23%), with the corresponding distributions being right‐skewed in both cases (Figure S1). Notably, per‐pseudochromosome comparisons detected in several cases extended variation (Figure 4), with each population displaying a higher median value for about half of the linkage groups.

### 3.5 Mitogenome assembly and haplotype network

**FIGURE 5:**
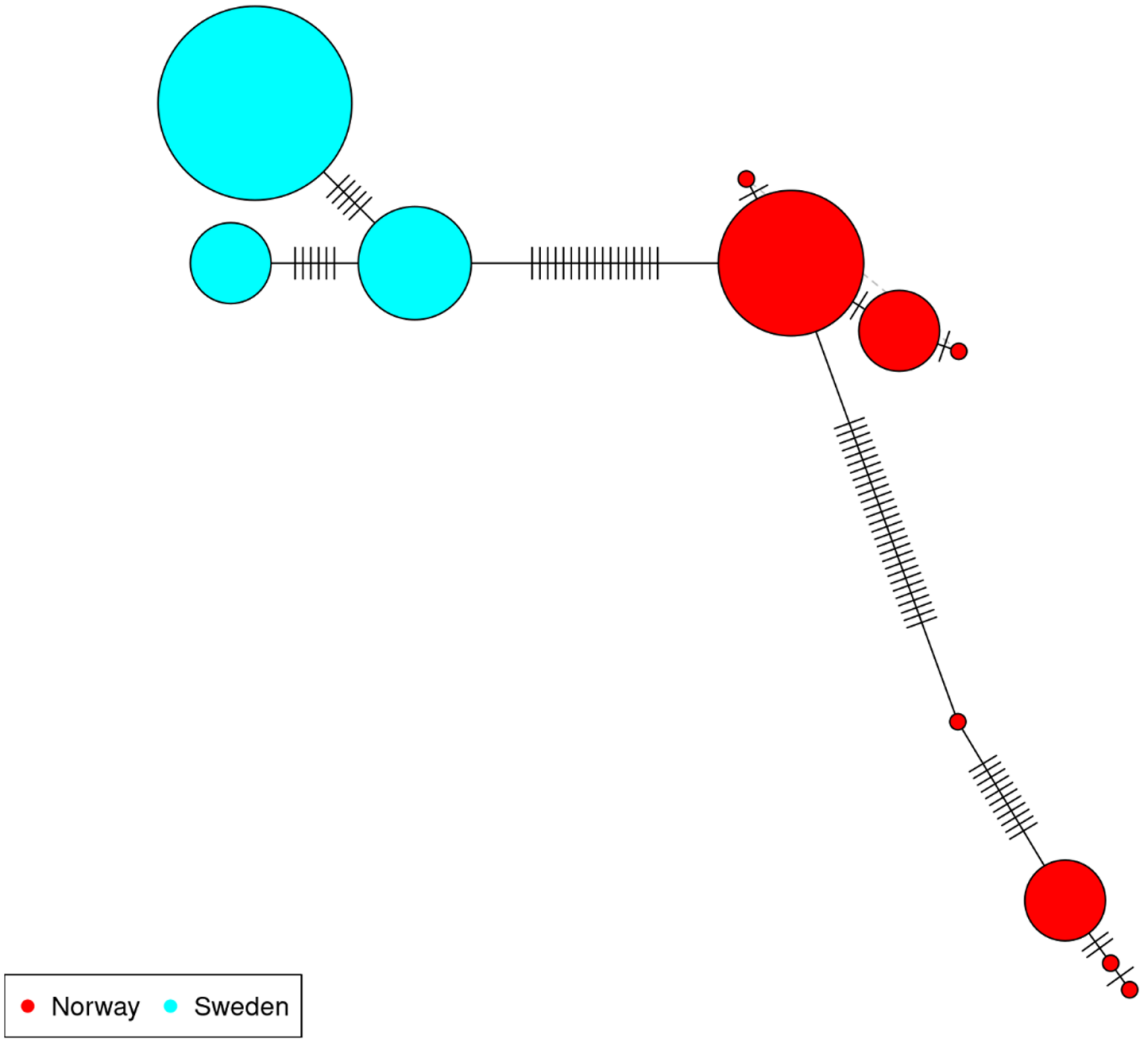
Haplotype network of the assembled mitochondrial genomes. Nodes are represented by circular discs with surface proportional to the number of individuals carrying the mt‐haplotype. Topological distances in the form of link lengths are proportional to the genetic distances represented by the number of mutation events (visualized as ticks on the links) separating the haplotype groups. Alternative links are indicated with dashed lines.

Mitochondrial genomes were de novo assembled for all 48 individuals. Their consensus sequence had a length of 16,658 nt and individual mt‐chromosome sizes ranged from 16,654 bp to 16,658 bp. Our analysis revealed 11 different mitochondrial haplotypes (Table S3) and the relationships between them were visualized through a haplotype network (Figure 5). As visualized by node topology, the Norwegian population seems to have a more extended diversity of mt‐genomes (8 haplotypes, maximum distance = 44 mutations) than the Swedish breeding nucleus (3 haplotypes, maximum distance = 11 mutations). Overall, 17 and 65 mutation events separated the two closest neighbouring and the two most distant haplotypes of the two populations respectively.

### 3.6 Detection of selective sweeps

**FIGURE 6:**
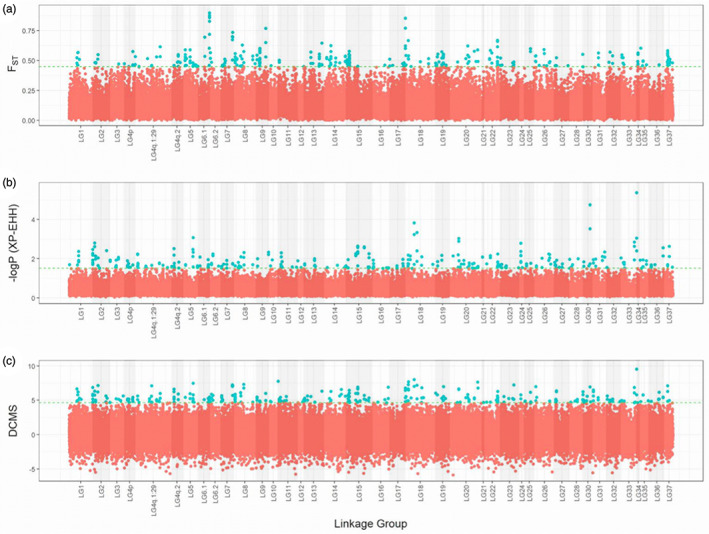
Manhattan plots showing selection metric values: (a) Weir and Cockerham's mean *F*_ST_, (b) negative log_10_ of the averaged *p*‐values of the XP‐EHH estimates, (c) DCMS values as defined in Equation (2). Points representing the 50 kb genomic windows are grouped within their respective pseudochromosomes and plotted along the horizontal axis based on physical position. Horizontal green lines correspond to the 99.5% percentile cut‐offs of each statistic and windows passing these cut‐offs are shown in blue.

The *F*_ST_ and XP‐EHH metrics used to detect selection signatures highlighted multiple genomic regions expected to have undergone selection in recent evolutionary time (post lineage formation). The 99.5% quantile cut‐off for *F*_ST_ was 0.449 (Figure 6a), while the same threshold for −logP(XP‐EHH) was 1.509 (Figure 6b). Genomic windows with DCMS values above 4.646 belonged to the top 0.5% of the empirical distribution (Table S4) and were regarded as candidate selection signals (Figure 6c, Figure S2).

**FIGURE 7:**
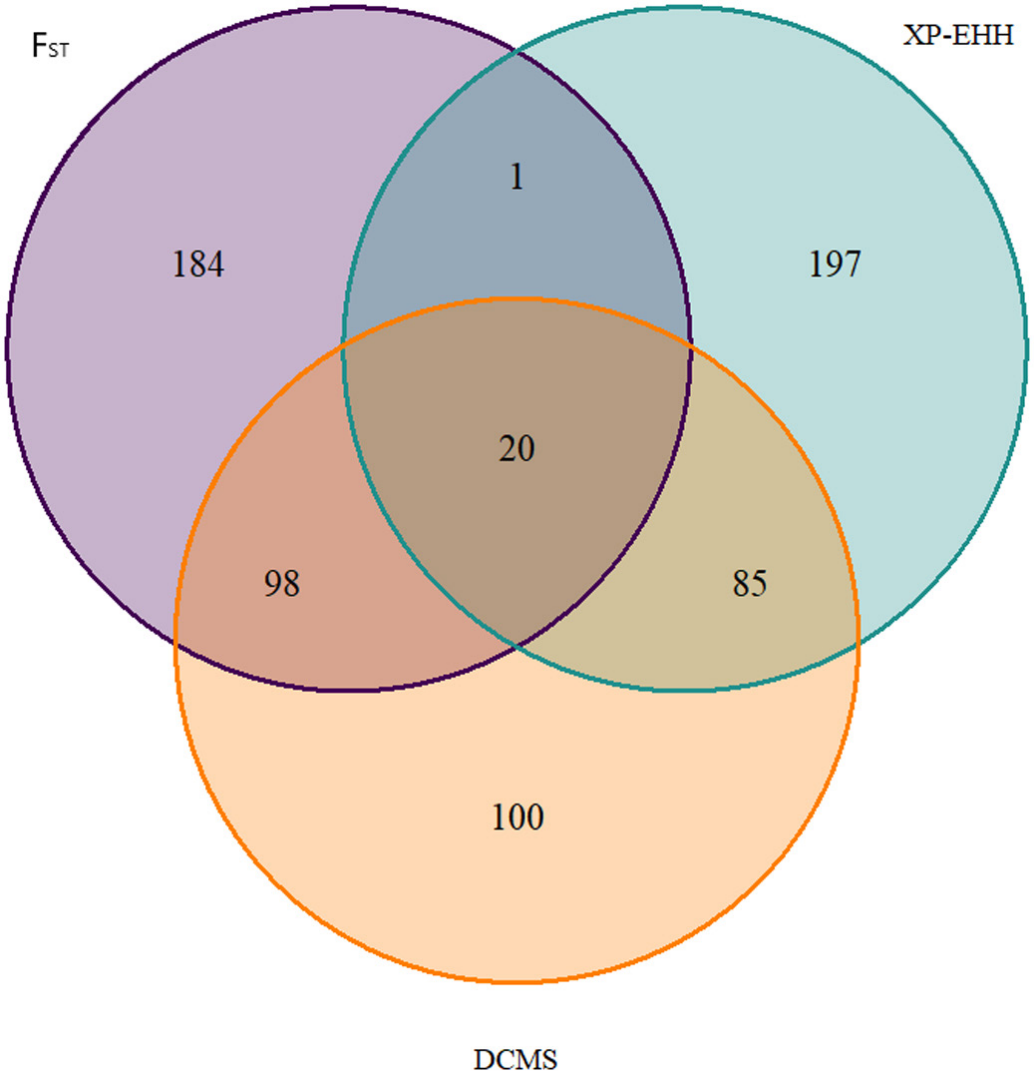
Venn diagram showing the counts of shared and unique hits of the three statistics *F*_ST_, XP‐EHH and DCMS used to identify genomic regions expected to have undergone positive selection.

Amongst the top 0.5% window sets of XP‐EHH and *F*_ST_, 21 common windows were found, while DCMS identified 20 of them in its own 99.5% quantile. Furthermore, DCMS detected 98 and 85 common windows with *F*_ST_ and XP‐EHH respectively (Figure 7).

### 3.7 Annotation and enrichment analysis of selection signatures

In total, 21 candidate regions belonging to the intersection of the *F*_ST_ and XP‐EHH hit sets were detected, with 25 genes found within them (Table 2). Out of those windows, 12 had a 50% overlap with another window, resulting in six 75 kb and nine 50 kb regions. Most genomic features extracted from the annotation file (GFF3) of the reference assembly were related to protein‐coding genes, while two of them were uncharacterized ncRNA genes. DCMS returned all but one (NC_036876.1: 18,600,000–18,650,000) of those 21 windows in its own 99.5% quantile.

**TABLE 2:** Genomic regions corresponding to the 21 windows derived from the intersection of the 99.5% quantile sets of *F*_ST_ and XP‐EHH. Overlapping windows are concatenated and physical ranges, linkage groups and genomic feature annotation information are provided for each region.

Linkage group	Window range (bp)	Size	Annotated gene/description
NC_036839.1	13,925,000–14,000,000	75 kb	LOC111974739/cordon‐bleu protein‐like 1 *slc38a11*/solute carrier family 38 member 11 LOC111974756/sodium channel protein type 2 subunit alpha‐like
NC_036843.1	6,800,000–6,850,000	50 kb	LOC111963015/uncharacterized ncRNA
NC_036844.1	25,025,000–25,100,000	75 kb	LOC111963657/C‐C motif chemokine 19 LOC111964090/PHD finger protein 24‐like
NC_036847.1	30,775,000–30,850,000	75 kb	–
NC_036848.1	3,300,000–3,350,000	50 kb	*mbnl3*/muscleblind‐like splicing regulator 3 LOC111967241/bromodomain‐containing protein 8 LOC111967242/DNA damage‐inducible transcript 4‐like protein LOC111967243/stimulator of interferon genes protein
NC_036848.1	24,800,000–24,875,000	75 kb	LOC111967632/protein diaphanous homolog 2‐like
NC_036854.1	53,600,000–53,650,000	50 kb	LOC111973584/cadherin‐7‐like
NC_036855.1	6,925,000–6,975,000	50 kb	*ttc37*/tetratricopeptide repeat domain 37 LOC111974700/arrestin domain‐containing protein 3
NC_036855.1	30,850,000–30,900,000	50 kb	*npffr1l2*/neuropeptide FF receptor 1 like 2 *hk2*/hexokinase 2
NC_036858.1	6,225,000–6,275,000	50 kb	LOC111978656/uncharacterized ncRNA
NC_036860.1	69,350,000–69,425,000	75 kb	LOC111980925/protein FAM171A2 LOC111980077/integrin alpha‐IIb‐like
NC_036862.1	31,600,000–31,650,000	50 kb	LOC111949543/endothelin‐converting enzyme‐like 1 LOC111982719/mucin‐2‐like
NC_036874.1	7,200,000–7,250,000	50 kb	LOC111958662/uncharacterized protein coding LOC111958663/SWI/SNF complex subunit SMARCC1‐like
NC_036876.1	6,525,000–6,600,000	75 kb	LOC111960328/ATP‐binding cassette sub‐family A member 1‐like
NC_036876.1	18,600,000–18,650,000	50 kb	LOC111960074/ELAV‐like protein 2

**FIGURE 8:**
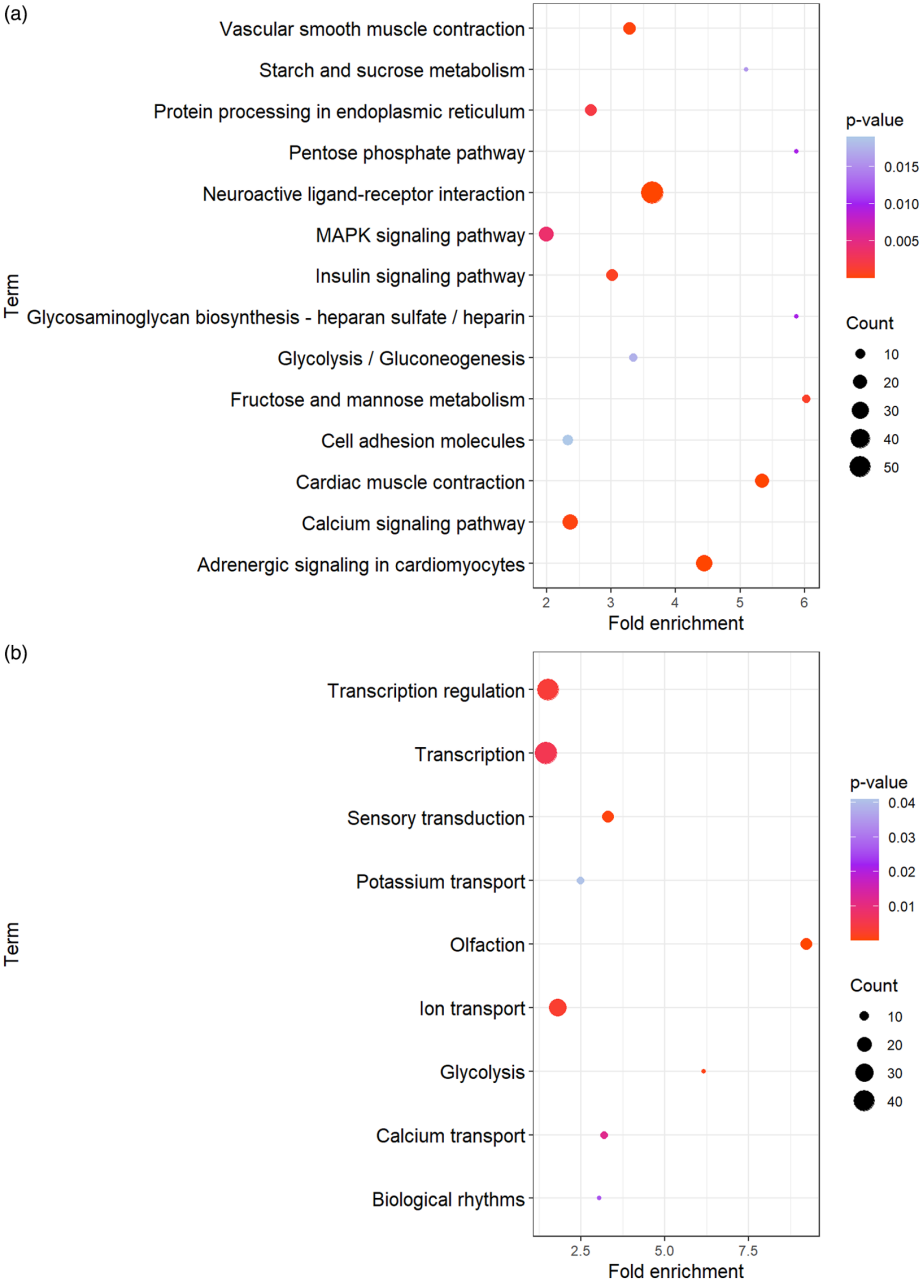
Visualizations of significantly enriched terms: (a) KEGG pathways, (b) UniProt functional annotation terms (biological‐process keywords). The surface area of each disk is proportional to the number of genes in the input list belonging to the term. Unadjusted significance level is indicated by the colour scale presented on the right side of each graph.

The top 303 (99.5% quantile) DCMS genomic windows (Table S4), corresponding to a total of 16.27 Mb on the reference assembly were included in the enrichment analysis. Overall, 16 KEGG pathways were detected (Table S5) with 14 of them being statistically significant (unadjusted *p <* 0.05) with an up to 6‐fold enrichment. The KEGG term ‘dre00051: Fructose and mannose metabolism’ was the one with the highest relative enrichment since it was 6.03 times overrepresented in the submitted gene set. The most significant KEGG pathway, ‘dre04080: Neuroactive ligand‐receptor interaction’, was also the one that included the most genes (55 gene IDs) in our input set and ranked seventh by fold of enrichment (Figure 8a).

Additional to KEGG pathways, 10 UniProt biological process keywords were highlighted (Table S6), of which nine were statistically significant. *KW‐0552 ~ Olfaction* was the term with the highest significance and relative enrichment. Furthermore, *KW‐0804 ~ Transcription* and *KW‐0805 ~ Transcription* regulation were the two significant terms with the largest number of genes in the hit list with 45 and 44 elements respectively (Figure 8b).

**FIGURE 9:**
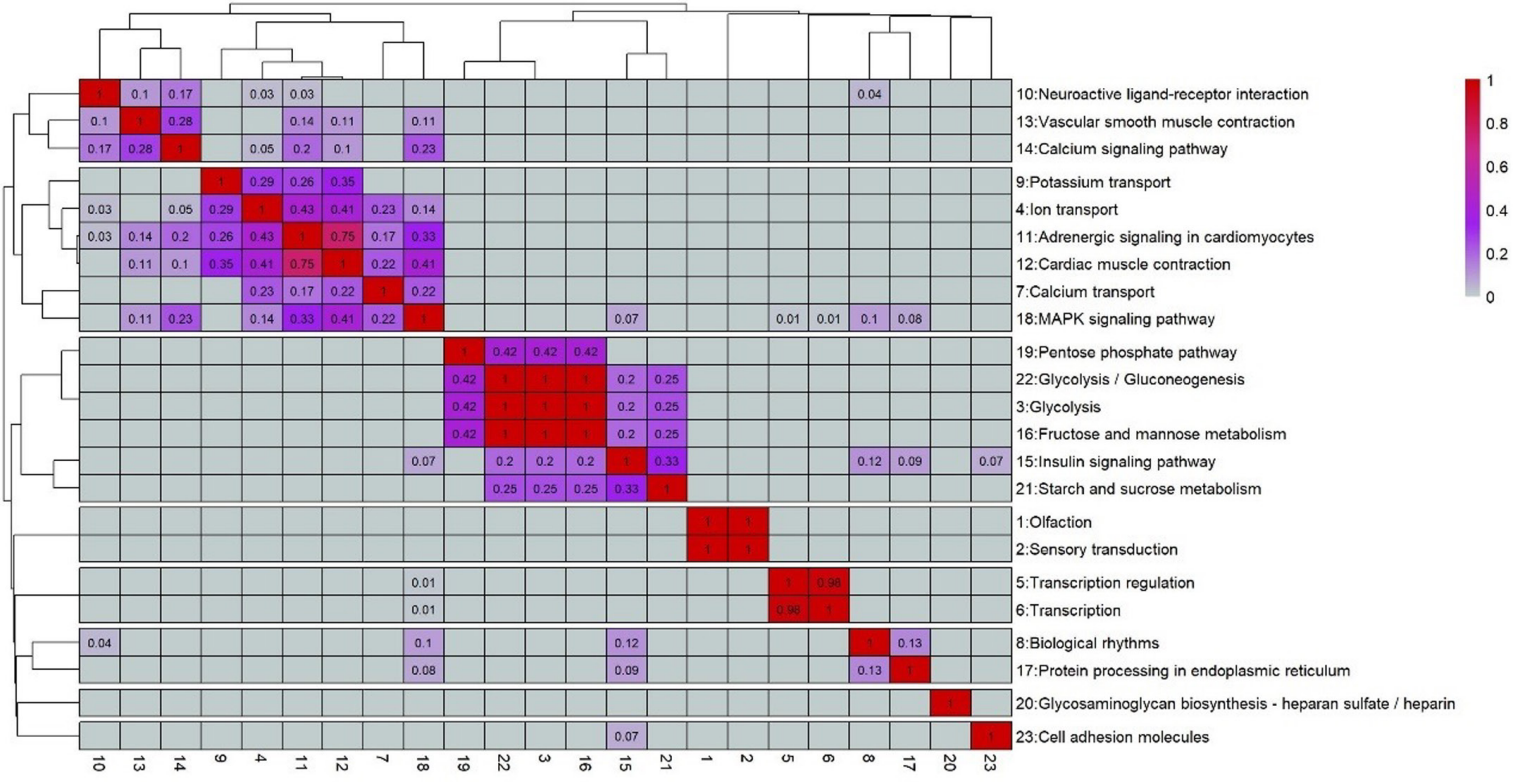
Clustered term‐similarity heatmap of the enriched KEGG pathways and UniProt keywords. Spaced row‐groups correspond to seven‐term clusters. Numeric values and colour of each cell correspond to the Jaccard index for each pairwise comparison. Empty cells indicate comparisons with a score of zero (A∩B=∅).

Finally, term‐similarity (Jaccard index defined in Equation 3) scores for each pairwise comparison between gene sets corresponding to different pathways and functional terms were visualized in the form of a clustered heatmap (Figure 9). The included dendrograms identified clusters of similar terms (or terms whose respective enriched gene sets contain common elements). Overall, those groups summarize biological functions that are mainly affiliated with ion transport, cardiovascular function, carbohydrate metabolic pathways, sensory perception and transcription.

## 4 DISCUSSION

Arctic charr is a salmonid of both commercial and ecological importance. Even though the value of genomics in both aquaculture and conservation settings is widely recognized, the availability of genomic resources for Arctic charr is still relatively limited. Our study reports the first whole‐genome re‐sequencing attempt on Nordic farmed Arctic charr. Sequencing of 48 broodfish from Sweden and Norway yielded a dense and high‐quality marker set that allowed the study of a series of aspects affiliated with population genetics. It has to be stressed, that in our study we used as a template for detecting SNPs the reference genome of the closely related species *Salvelinus malma* or a hybrid between this and Arctic charr as no publicly available genome for the latter is currently available. Unavoidably, the above could have resulted in missing unique genomic features that are to be found in pure Arctic charr. However, the obtained alignment rate was satisfactory (~85%) suggesting that the currently available reference genome is a suitable choice for genetic studies in Arctic charr.

### 4.1 Genetic structure and levels of inbreeding

As shown by the genetic relationship matrix, no closely related individuals were used which suggests that the studied animals were reasonably representative of the entire studied populations. Substantial genetic differentiation between the two populations was suggested by the pairwise *F*_ST_ analysis (~0.12), which was also confirmed through PCA, where the two populations were easily distinguishable. Additionally, admixture analysis showed that the optimal number of clusters for the metapopulation was two and as expected, no hybridization of the two stocks was evident. Those results appear to agree with the fact that the two populations have quite distinct origins, with the Norwegian having an anadromous background, while the Swedish originating from a landlocked population. Notably, no apparent evidence of admixture was found for the Norwegian population, even though it originates from three distinct geographical locations (Hammerfest and Svalbard in Norway, Iceland). A possible explanation could lie in the fact that due to artificial selection, the current gene pool represents mainly only one of the founding populations.

Nevertheless, the above hypothesis would have to be tested in a future study that should also include animals from the aforementioned geographical locations. Furthermore, it would be worth also to point out that the constructed haplotype network of our study from each animal mitogenome clearly supported the existence of a more diverse maternal gene pool in the case of the Norwegian population. However, this could also be due to the landlocked versus anadromous background of the two populations.

Overall, the observed heterozygosity was almost identical between populations (~0.29) and close to the values previously reported for the Swedish one that relied on low‐density genotyping (Palaiokostas et al., 2022). The estimates of historical *N*_e_ trends also appeared comparable. A similar, linear descending pattern through time was evident for both populations, with estimates for the Swedish stock being slightly but stably higher than the ones of the Norwegian. As a general rule of thumb, an *N*_e_ above 100 is encouraged to retain a population's fitness and minimize inbreeding depression. However, *N*_e_ sizes well below 50 have been recently reported for several farmed fish species like coho salmon (*Oncorhynchus kisutch*) (Barria et al., 2019), carp (*Cyprinus carpio*), seabream (*Sparus aurata*), seabass (*Dicentrarchus labrax*) and turbot (*Scophthalmus maximus*) (Saura et al., 2021; Villanueva et al., 2022). Nevertheless, we need to stress that significant standard errors accompanied our *N*_e_ estimates. Therefore, it is not possible to draw reliable inferences regarding the exact trend of *N*_e_ over the past generations.

A complementary analysis, on the other hand focusing on linkage disequilibrium distribution, showed that LD declined more rapidly in the Norwegian population. However, overall, the LD distribution for both populations was in line with similar studies in other salmonid species. More specifically, compared to an average LD of 0.09 and 0.12 at 100 kb for the Norwegian and Swedish populations of our study, the corresponding average LD for anadromous trout and coho salmon was approximately 0.15 and 0.16 respectively (Barria et al., 2019; Cádiz et al., 2021). Notably, markers in a 50 k SNP array for Norwegian and Swedish Arctic charr would have on average, an *r* ^2^ of 0.14 with their closest neighbouring site (assuming a distance of 40 kb), which should suffice for future applications of genomic selection (Meuwissen et al., 2001).

The Swedish population also had higher estimates of genomic inbreeding since more and longer homozygous genomic regions were revealed for its individuals by the ROH analysis. In particular, the obtained inbreeding estimate for the Swedish population was higher (8.66%) compared to a pedigree‐based estimate (5.8%) of a previous study (Palaiokostas et al., 2021). It should be stressed that the latter, by default, assumed that all the animals in the base population were unrelated.

At first notice, the fact that a higher inbreeding estimate was found for the Swedish population is unexpected since the selection strategy used in the Norwegian farm (mass selection—no pedigree records) does not take into account the relation level of the breeding pair and thus can result in higher inbreeding rates (Bentsen & Olesen, 2002; Khaw et al., 2014). Though, it is worth noting that the Swedish breeding programme is older (nine generations) than the broodfish of the Norwegian farm (seven generations). It is also worth to mention that a relatively small number of families have been used so far in the Swedish breeding programme. Moreover, the initial level of relatedness between the individuals of the base population was probably higher in the case of the Swedish population because this consisted of fish from only one lake (Hornavan) compared to three different and geographically isolated populations—origins in the Sigerfjord farmed stock which was also of anadromous background. Overall though, we need to mention that the obtained inbreeding levels for both populations based on ROH were substantially lower compared to documented ones from breeding programmes of coho salmon and rainbow trout, where while inbreeding exceeded 0.10 no signs of inbreeding depression were reported (D'Ambrosio et al., 2019; Yoshida et al., 2020).

### 4.2 Signatures of selection

Several genomic regions with a high probability of having been subjected to historical selection events were detected in our study. Some individual signals were placed in/or cover genes that indicated a very specific selective process such as the top DCMS hit (interferon‐inducible very large GTPase 1‐like) which might be a result of pressure due to pathogenic agents (Kim et al., 2012; Meunier & Broz, 2016; Pilla‐Moffett et al., 2016). Notably, genomic regions harbouring genes with immunity‐related functions have been recently pinpointed as putative selection signatures through the application of high‐density genotyping technologies in various farmed fish like Atlantic salmon (*Salmo salar*) (Gutierrez et al., 2016; López et al., 2019), coho salmon (*Oncorhynchus kisutch*) (López et al., 2021), rainbow trout (*Oncorhynchus mykiss*) (Cádiz et al., 2021), tambaqui (*Colossoma macropomum*) (Agudelo et al., 2022) and Nile tilapia (*Oreochromis niloticus*) (Cádiz et al., 2020). Such pattern, consistent across several different species, could be influenced by the fact that usually farmed fish are either directly selected for traits associated with disease resistance or due to the exposure to high levels of pathogens, strong selective pressure is applied in those genomic regions (López et al., 2015; Zueva et al., 2014). Nevertheless, since the most likely factor behind the suggested selective sweeps in our study is the anadromous versus the landlocked background of the two populations the candidate selective sweep could also reflect the exposure to pathogens present in various environments versus the relatively limited diversity of infectious agents in a single lake.

Regarding the rest of the yielded set of genes that were located in putative selective sweeps, additional biological pathways subjected to selection pressure were suggested. The most noteworthy of them were affiliated with cardiovascular function, ion transport, transcription, blood coagulation, carbohydrate metabolism and processing of environmental stimuli. As already mentioned, a major factor that could explain the identified selection signatures and their corresponding biological functions is the anadromous background of the Sigerfjord stock (Rikardsen et al., 1997; Wietrzyk‐Pełka & Węgrzyn, 2020) and the fact that it is being reared in brackish water compared to the Swedish stock where only fresh water is used. In particular, genomic regions harbouring genes involved in biological processes associated with osmoregulation and water salinity tolerance (e.g. ion transport; potassium transport; calcium transport) were suggested as putative selection signatures. Consistently to our results, previous studies comparing anadromous and landlocked salmonids using high‐density genotyping technologies have also detected selection signatures in genomic regions where genes associated with osmoregulation and salinity tolerance reside (Cádiz et al., 2021; Kjærner‐Semb et al., 2021).

Current knowledge suggests that the migratory behaviour of fish, such as anadromy in salmonids, results in selective pressure on genomic regions associated with olfaction and sensory perception in general (Gao et al., 2021). The above is most likely due to the fact that navigation for natal homing of migrating individuals relies on the detection and processing of environmental stimuli such as odours (Bett & Hinch, 2016; Døving et al., 1985; Ueda et al., 2016; Wisby & Hasler, 1954; Yamamoto et al., 2010). Furthermore, migration also requires long‐distance upstream swimming towards spawning grounds and thus, such stocks are additionally subjected to selection for swimming ability, aerobic capacity and circulatory system robustness. Therefore, the keyword ‘*KW‐0090 ~ Biological rhythms*’ could also be associated with anadromy since fish migration is a rhythmic behaviour (Brüning et al., 2015; Lowe, 1952).

Another possible explanation for some of the detected signals of historical selection appears to be domestication. Terms affiliated to heparan/blood coagulation, sensory perception (mainly olfaction), transcription and energy metabolism have been previously reported in studies aiming to uncover selective sweeps in genomes of domesticated animals (Liu et al., 2017; Qanbari et al., 2014). Artificial selection for production traits in Atlantic salmon is known to alter the genomic and expression profile of genes involved in transcription and metabolic pathways (Bicskei et al., 2014) since its main aim is to increase the efficiency of feeding and growth rate. Furthermore, wild salmonid populations follow a carnivorous diet and hence, adaptation to a plant‐based feed that is nowadays common in aquaculture and which is richer in carbohydrates could have led to the respective sweeps. Similar sweeps have been identified in canine genomes as a consequence of adaptation to a starch‐rich diet (Axelsson et al., 2013). Moreover, domesticated populations of teleost species are known to have different heart morphology (Poppe et al., 2003), reduced aerobic capacity and altered cardiovascular function (Zhang et al., 2016) compared to fish from natural stocks, facts that might be associated with the yielded gene enrichment results of our study concerning such biological functions.

## 5 CONCLUSION

Whole‐genome re‐sequencing of 48 farmed Arctic charr broodfish derived from Sweden and Norway yielded dense genomic information in the magnitude of millions of genetic markers. The conducted analyses of the nuclear genome variation provided insights on genetic diversity, levels of inbreeding and historical demography. At the same time, genome‐wide scanning approaches revealed putative selection signatures and their associated biological functions. The interpretation of the latter allowed hypotheses formation regarding the context of past selection events attributed primarily to the anadromous or landlocked background and secondarily on domestication of the studied populations.

## CONFLICT OF INTEREST STATEMENT

The authors declare no conflict of interest.

## Supporting information


Data S1:

## Data Availability

Sequenced reads in the form of fastq files were deposited to the National Centre for Biotechnology Information (NCBI) repository and are publicly available under the project ID *PRJNA860083*. Moreover, the entire code that was used to perform the analysis is available in the form of an R Markdown file in the supplementary under the name *Arctic_charr_WGS_code.Rmd*. Finally, to facilitate reproducibility *yaml* files containing complete information about each computational environment (versions of each package and its dependencies) that was used for each of the conducted analyses are available in the supplementary. Detailed information about each of the aforementioned files and the corresponding performed analysis can be found in (File S1).
