# Supplementary material for: Whole‐genome re‐sequencing provides key genomic insights in farmed Arctic charr (Salvelinus alpinus) populations of anadromous and landlocked origin from Scandinavia
Source: Evol Appl. 2023 Feb 27;16(4):797–813. doi: 10.1111/eva.13537 (PMC10130564; doi:10.1111/eva.13537)
Supplement: Supplementary file 1 — Data S1: [file EVA-16-797-s001.zip › EVA_13537_File S1.docx]

**Table S7.** Role of supplementary files that can be used to replicate the conducted analysis

| **File** | **Type of information** |
| --- | --- |
| full_acwgs.Rmd | Documents the entire range of conducted analysis |
| filters.yml | Computational environment that was used during QC |
| aligners.yml | Computational environment that was used for aligning the sequenced reads to the reference genome |
| biotools.yml | Computational environment that was used for adding read group tags, filtering SNPs, ROH analysis, admixture analysis, GRM and scanning for selective sweeps |
| genotypers.yml | Computational environment that was used for detecting SNPs |
| annotation.yml | Computational environment that was used for annotation and enrichment analysis |
| assemblers.yml | Computational environment that was used for assemblying mt-genomes |
