# Supplementary material for: Whole‐genome re‐sequencing provides key genomic insights in farmed Arctic charr (Salvelinus alpinus) populations of anadromous and landlocked origin from Scandinavia
Source: Evol Appl. 2023 Feb 27;16(4):797–813. doi: 10.1111/eva.13537 (PMC10130564; doi:10.1111/eva.13537)
Supplement: Supplementary file 1 — Data S1: [file EVA-16-797-s001.zip › EVA_13537_Missing_captions.docx]

**Captions**

File S1. Detailed information on the role of each supplementary file in the data analysis.

File S2. MultiQC report part I.

File S3. MultiQC report part II.

Figure S1. Range of the genomic inbreeding coefficient F_ROH_ in the Swedish and Norwegian populations of farmed Arctic charr.

Figure S2. A. Scatterplot depicting the cross-population extended haplotype homozygosity [-logP(XP-EHH)] on the x-axis and the F_ST_ index on the y-axis. B. QQ-plot between the de-correlated composite of multiple metrics (DCMS) and the theoretical quantiles of the standard normal distribution.

Table S1. Predictions of effective population size (Ne) of Swedish Arctic charr across past generations.

Table S2. Predictions of effective population size (Ne) of Norwegian Arctic charr across past generations.

Table S3. Mitochondrial haplotypes for each of the sequenced Arctic charr.

Table S4. De-correlated composite of multiple metrics (DCMS) estimates across the genome.

Table S5. Detected KEGG pathways in genomic regions under selection.

Table S6. UniProt biological process keywords in genomic regions under selection
