# Supplementary figures and images for: Whole‐genome re‐sequencing provides key genomic insights in farmed Arctic charr (Salvelinus alpinus) populations of anadromous and landlocked origin from Scandinavia
Source: Evol Appl. 2023 Feb 27;16(4):797–813. doi: 10.1111/eva.13537 (PMC10130564; doi:10.1111/eva.13537)

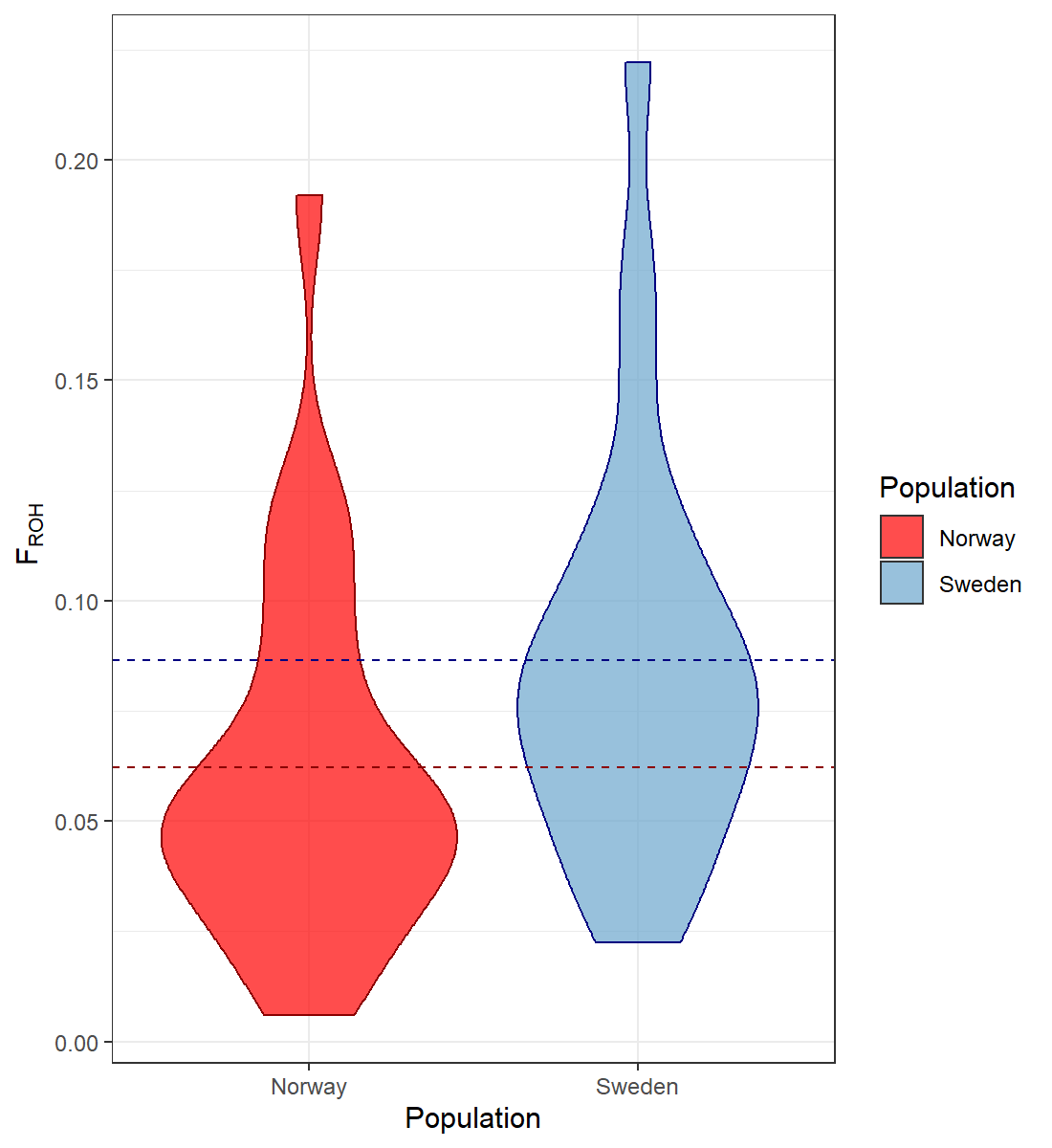

Supplement: Supplementary file 1 — Data S1: [file EVA-16-797-s001.zip › EVA_13537_Figure_S1.png]

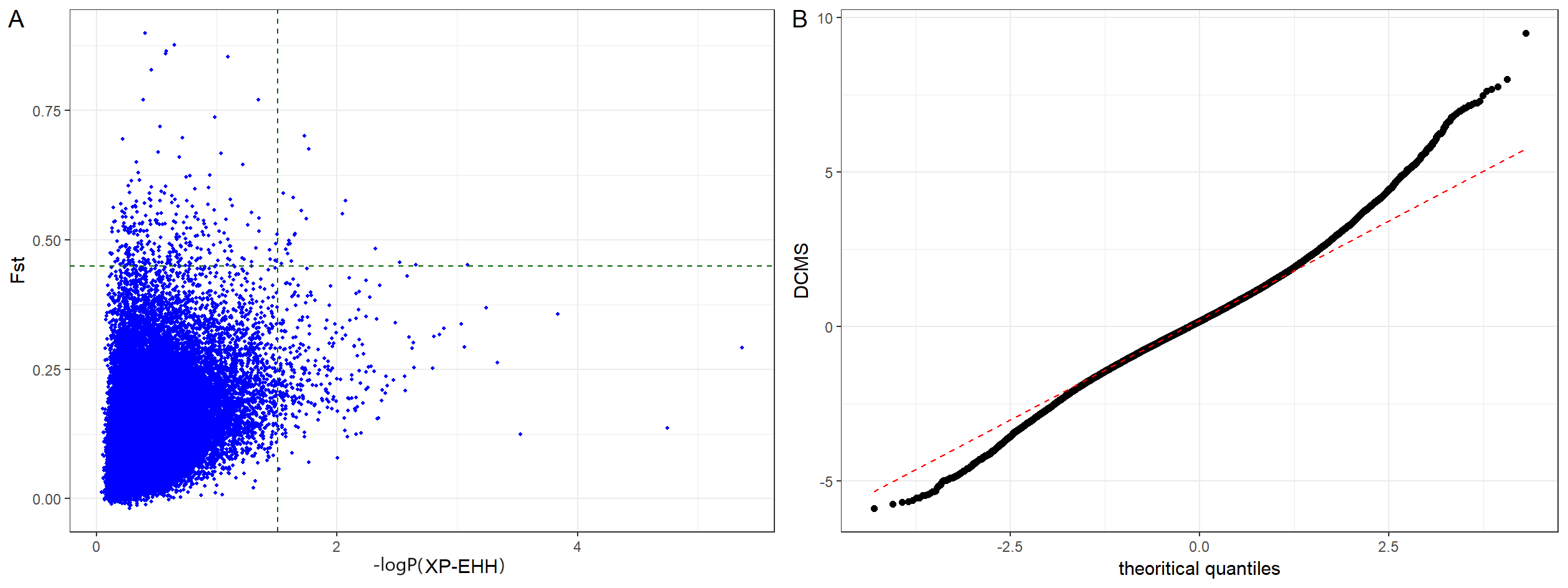

Supplement: Supplementary file 1 — Data S1: [file EVA-16-797-s001.zip › EVA_13537_Figure_S2.tiff]
